# *Eigeneyes*: A statistical model of whole-eye geometry and its applications

**DOI:** 10.21203/rs.3.rs-10081326/v1

**Published:** 2026-09-14

**Authors:** Estela Vadillo, Álvaro de la Peña, Javier Rodríguez-Sánchez, Alberto de Castro, Susana Marcos, Eduardo Martínez-Enríquez

**Affiliations:** 1 Consejo Superior de Investigaciones Científicas (CSIC), Calle Serrano 121, Madrid, 28006, Spain; 2 Flaum Eye Institute, University of Rochester, 500 S Clinton Avenue, Rochester, NY 14620, United States; 3 Center for Visual Science, University of Rochester, Rochester, NY, United States

## Abstract

Modeling the three-dimensional geometry of the optical surfaces of the human eye has important applications, including the study of myopia and presbyopia, as well as the planning of cataract surgery. In this paper, we present the *Eigeneyes*, a statistical shape model of the complete three-dimensional geometry of the human eye based on principal component analysis (PCA). The model was constructed from 572 Optical Coherence Tomography eye scans in which the cornea, crystalline lens, and retina were visible. *Eigeneyes* captures geometric variability across ocular structures using a small number of coefficients that represent global modes of variation. *Eigeneyes* achieved a statistically significantly lower reconstruction error than other state-of-the-art methods while using fewer parameters. Furthermore, the *Eigeneye* coefficients were modeled within a multivariate probabilistic framework, enabling the generation of realistic synthetic eyes conditioned on age and/or axial length.

## 1 Introduction

The human eye is a complex optical system in which the cornea and the crystalline lens work in a coordinated manner to focus light onto the retina, where photosensitive cells convert light into neural signals. Consequently, the geometry, relative position, and alignment of these structures play a key role in visual performance and in the development of refractive errors and ocular pathologies.^1, 2^. Numerous highly prevalent visual conditions, such as myopia, presbyopia and glaucoma, are associated with geometric and optical changes in the eye. Therefore, their study and treatment would benefit from accurate and personalized models capable of describing these changes^3^. Cataracts, on the other hand, are characterized by a loss of transparency of the crystalline lens and require its replacement with an intraocular lens (IOL) through surgery, which is one of the most frequently performed surgical procedures worldwide. Visual quality after cataract surgery largely depends on the accurate preoperative selection of the IOL power, which in turn depends on the final position of the implanted lens. The estimation of this position is usually based on the patient’s preoperative ocular geometry and would therefore benefit from personalized geometrical models^4, 5^.

Traditionally, geometrical models of the human eye have described anatomical components using sets of parameters, such as radii of curvature, asphericities, thicknesses, and axial distances. Classical examples include the Gullstrand model^6^ and subsequent schematic models that incorporated aspheric surfaces to improve optical accuracy^7–9^. Although these models have been widely used in physiological optics, ophthalmology, and computational simulations, they present two main limitations: (i) they often require a large number of parameters to accurately describe the full ocular geometry^10^; (ii) classical schematic eye models often rely on simplified or partially decoupled descriptions of ocular components (e.g., univariate linear dependencies of geometric parameters on age or refraction), which do not fully capture the complex multivariate relationships between structures like the cornea and crystalline lens^11^. In reality, ocular surfaces do not vary independently, but exhibit strong geometric correlations driven by development, growth, and biomechanics^12^. Consequently, modeling each structure separately limits the ability to describe the eye as an integrated system and can lead to inconsistencies when independently derived geometric parameters are combined.

High resolution imaging techniques have represented a major advance in the characterization of the in vivo human eye. In particular, optical coherence tomography (OCT), introduced in the early 1990s^13^, enables three-dimensional imaging of the cornea, crystalline lens, and retina with micrometer scale resolution and acquisition times compatible with routine clinical use. The availability of large amounts of detailed volumetric data of the entire eye has opened new possibilities for the development of more accurate geometric models^14^.

In this context, statistical modeling approaches based on principal component analysis (PCA) have emerged as an effective strategy to reduce data dimensionality while capturing the main modes of inter subject variability. PCA was popularized in computer vision through the *Eigenfaces* framework^15^, and has been previously applied to ocular modeling. For instance, Rozema et al.^16^ incorporated PCA into schematic eye models to describe population variability using biometric and optical parameters, while Rodriguez et al.^17^ applied PCA to Zernike-based representations of corneal elevation maps . However, in these approaches, variability is defined in terms of a selected set of parameters or is limited to specific structures (e.g., corneal shape in *Eigencorneas*), which constrains the range of shapes that can be represented and does not fully capture how different parts of the eye vary together.

More recent works have applied PCA directly to volumetric imaging data, rather than to geometric parameters, to model individual ocular structures, such as the *Eigenlenses* model for the crystalline lens^18^. The *Eigenlenses* model, constructed from OCT images of isolated lenses^19^, has been used in multiple applications, such as the study of crystalline lens morphological changes associated with myopia^3^, the estimation of the complete lens geometry (including its periphery) *in vivo* from partial OCT data acquired through the pupil^20^, the improvement of the prediction of the IOL position^21^ and tilt after cataract surgery using machine learning techniques^22^, as well as the *in vivo* estimation of the biomechanical parameters of the lens^23^. The model has been validated both *ex vivo* and *in vivo*^24, 25^. Nevertheless, the *Eigenlenses* approach is limited to the crystalline lens and does not provide a framework for jointly modeling the geometry of the entire eye.

In this paper we propose the *Eigeneyes*, a statistical framework designed to represent the complete three-dimensional geometry of the human eye. The model is constructed from the three-dimensional geometries of 572 eyes obtained from OCT measurements acquired using a commercial system (IOL Master700, Carl Zeiss Meditec AG, Jena, Germany). Using *Eigeneyes*, each eye is represented as the sum of a mean shape and a linear combination of global modes of variation that simultaneously affect all relevant ocular structures. In contrast to conventional independent parameterizations, these modes capture coupled variations in the shape, tilt, and relative position of the cornea, crystalline lens, and other anatomical components, reflecting the inherently interconnected nature of the ocular optical system. We compare the accuracy and compactness of *Eigeneyes* with other representation methods. We also model the *Eigeneye* coefficients, which weight the contribution of each mode of variation, using a joint multivariate Gaussian distribution. This probabilistic formulation enables sampling consistent sets of coefficients and, consequently, the generation of realistic synthetic eye geometries for a given age and/or axial length. We show that the geometrical changes with age and axial length of these synthetic eyes are consistent with those reported in the literature, with the added advantage of being able to decouple the effects of both variables. This allows us to generate an arbitrary number of realistic models with important applications in the study of myopia and presbyopia. Furthermore, the compaction ability of the representation will provide advantages in estimation problems, such as the IOL position estimation.

## 2 Methods

### 2.1 Dataset

The dataset, partially used in a previous publication^22^, consists of 733 eyes from 486 patients that were acquired from five institutions: the Flaum Eye Institute, University of Rochester (Rochester, NY, EE.UU); the Department of Ophthalmology, Baylor College of Medicine (Houston, TX, EE.UU); the Hospital Universitario Fundación Jiménez Díaz (Madrid, Spain); the LV Prasad Eye Institute (Hyderabab, India); and the Instituto de Óptica, CSIC (Madrid, Spain). Exclusion criteria included a history of previous ocular surgery, such as corneal refractive procedures (e.g., LASIK), pregnancy, and designation of vulnerable-groups. The measurements were obtained using the commercial swept-source OCT system IOLMaster700 (Carl Zeiss Meditec AG, Jena, Germany)^26^. For each eye, IOLMaster700 was used to acquire OCT scans along six evenly spaced meridians, separated by 30 degrees, covering a 6 mm lateral extent in which the cornea, the iris, the crystalline lens and the retina, were visible. All measurements were performed with natural, non-dilated pupils. Since iris pigmentation limits the visibility of the lens, only measurements with pupil diameters greater than 3 mm were included. The resulting dataset included a total of 572 eyes from 392 subjects. The mean age was 65 years, (standard deviation = 14 years), ranging from 21 to 92 years. The mean axial length was 23.77 mm (standard deviation = 1.74 mm), with values ranging from 19.53 mm to 32.82 mm. Of these, 305 were right eyes and 267 were left eyes. Regarding sex distribution, 282 eye were from women and 193 were from men, while for 81 eyes no information about sex was available.

All procedures followed for obtaining the measurements satisfy the principles of the Declaration of Helsinki, ensuring that the measurements met ethical, scientific, and participant well-being standards. Ethical approval was granted by the Ethics Committee of University of Rochester Medical Center, Baylor College of Medicine, Fundación Jiménez Díaz, LV Prasad Eye Institute and Consejo Superior de Investigaciones Científicas (CSIC). Each subject signed an informed consent form approved by the ethics committee of the various institutions involved in the data collection for this project.

### 2.2 Obtaining the 3-D shape of the eyes

**Figure 1.**
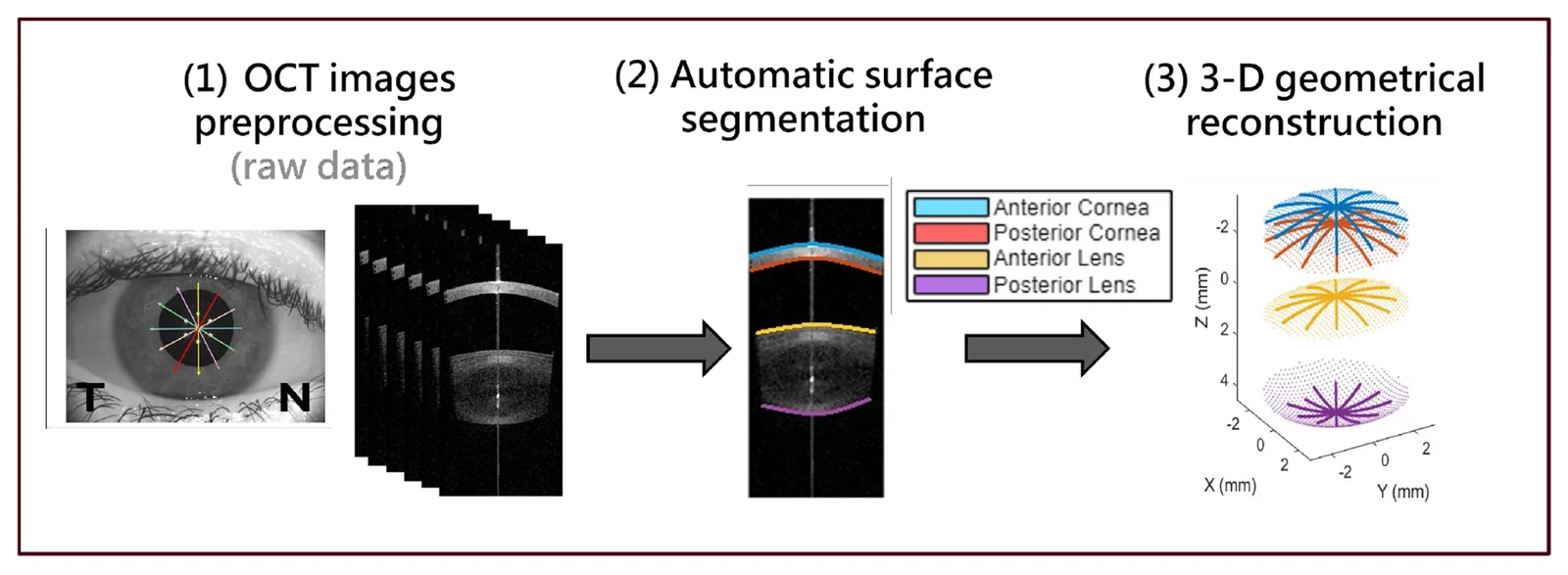
Main processing pipeline for obtaining the three-dimensional geometry of the eye from anterior segment OCT images. (1) Preprocessing of raw OCT B-scans from the IOLMaster700. (2) Automatic segmentation of the anterior and posterior surfaces of the cornea and crystalline lens using a deep learning-based algorithm. (3) Three-dimensional reconstruction of the ocular structures from six registered meridians, followed by surface smoothing through Zernike polynomial fitting and extrapolation to a 6 mm pupil diameter. The retina is not shown for visualization purposes.

Figure 1 shows the main steps for obtaining the 3-D geometry of the eye from anterior segment OCT scans, which have been partially reported in previous publications^14, 22^. Briefly, these steps are: (1) OCT image preprocessing; (2) automatic segmentation of the anterior and posterior surfaces of the cornea and crystalline lens, and of the retina; and (3) 3-D shape reconstruction.

In the preprocessing step, images were cropped, rotated by 90°, and resized to ensure consistent orientation, remove irrelevant regions, and standardize input dimensions for subsequent analysis. Then, a segmentation algorithm based on deep learning (Feature Pyramid Network with a pretrained ResNet-34 encoder)^14^ was used to extract the relevant ocular structures. The 3-D shape reconstruction involved converting the segmented surfaces from pixels to millimeters using calibration data (i.e., the experimentally determined pixel size), correcting optical distortions, and registering the data from the six meridians into a single coordinate system. In addition, noisy data were filtered by removing the meridian that deviated most from the median of the six meridians, resulting in 3-D point clouds representing the cornea, lens, and retina. These point clouds were fitted with Zernike polynomials up to the 4th order (15 coefficients) for the cornea and the 2nd order (6 coefficients) for the lens to obtain smooth surface representations (see Section 4 for a discussion on the selection of the number of Zernike coefficients). The resulting representation was used to extrapolate the surfaces of the crystalline lens to a 6 mm pupil diameter when the pupil size was smaller. This extrapolation ensured a common radial domain of 6 mm across all eyes and structures. The retina was modeled as a planar surface, such that it does not contribute to shape variability in the *Eigeneyes* representation but serves only as a geometric reference for registering all eyes.

### 2.3 *Eigeneyes* basis representation

To obtain the *Eigeneyes* representation, a principal component analysis (PCA) was performed on the 3-D reconstruction of the ocular surfaces. Each 3-D eye comprised five surfaces: the anterior and posterior cornea, the anterior and posterior lens, and the retina. All surfaces were reconstructed and sampled on a common uniform X–Y grid with *P* points, with Z coordinates representing elevations obtained from the Zernike polynomials fitted to each surface. To allow consistent comparison across eyes, all eyes were aligned with the retina positioned on the *Z* = 0 plane as a reference.

Each eye was represented by a vector of length 5*P*, with *P* = 22020, obtained by concatenating the coordinates of the five ocular surfaces evaluated on the common X–Y grid. Thus, for each eye *n* ∈ {1, …, 572}, three coordinate vectors were defined:

$$x_{n}\in {\mathbb{R}}^{5P}\text{:}\;\text{vector with the X coordinates}(P\;\text{points}),\;\text{repeated for each of the}\;S=5\;\text{surfaces}.$$

$$y_{n}\in {\mathbb{R}}^{5P}\text{:}\;\text{vector with the Y coordinates}(P\;\text{points}),\;\text{repeated for each of the}\;S=5\;\text{surfaces}.$$

$$z_{n}\in {\mathbb{R}}^{5P}\text{:}\;\text{vector with the Z coordinates of the five ocular surfaces:}$$

$$z_{n}=\left[\begin{array}{c} z_{n}^{\left(1\right)}\\ z_{n}^{\left(2\right)}\\ z_{n}^{\left(3\right)}\\ z_{n}^{\left(4\right)}\\ z_{n}^{\left(5\right)} \end{array}\right]\in {\mathbb{R}}^{5P}$$

where $z_{n}^{\left(s\right)}\in {\mathbb{R}}^{P}$ represents the Z-axis values corresponding to the surface *s* of eye *n*, ordered as: *s* = 1 for the anterior cornea, *s* = 2 for the posterior cornea, *s* = 3 for the anterior lens, *s* = 4 for the posterior lens, and *s* = 5 for the retina.

In order to construct the *Eigeneyes* basis, the following steps were followed:

#### 1. Mean eye calculation.

After spatial registration at the retina, the *mean eye shape* was computed as the pointwise average of all eyes, yielding a 3-D model that represents the average ocular geometry in the dataset:

(1) $$\bar{z}=\frac{1}{N}\sum_{n=1}^{N}z_{n}\in {\mathbb{R}}^{5P},$$

where *N* denotes the number of eyes in the dataset. This average model acts as a reference for quantifying morphological variability across the population.

#### 2. Residual calculation.

For each eye *n*, the residues were calculated as the deviation from the mean shape

(2) $$\Delta_{n}=z_{n}-\bar{z},\;n=1,\ldots ,N,$$

where $\bar{z}$ denotes the mean eye shape.

#### 3. Computation of the covariance matrix of the centered data matrix.

The residual vectors of all eyes were included into the centered data matrix

(3) $$D=\left[\begin{array}{llll} \Delta_{1} & \Delta_{2} & \cdots & \Delta_{N} \end{array}\right]\in {\mathbb{R}}^{5P\times N}.$$

The sample covariance matrix was calculated as

(4) $$C=\frac{1}{N-1}{DD}^{⊤}\in {\mathbb{R}}^{5P\times 5P}.$$

#### 4. Principal component extraction.

The principal components, which we refer to as *Eigeneyes*, were obtained from the following eigenvalue problem:

(5) $$Ce_{k}=\lambda_{k}e_{k},$$

where **e**_*k*_ and *λ_k_* are the *k*-th eigenvector (*Eigeneye*) and eigenvalue, respectively. Because the dimensionality of the eye shape vectors (5*P*) was substantially larger than the number of samples (*N*), PCA was implemented using the compact singular value decomposition (SVD) formulation for computational efficiency. The *Eigeneyes* represent the dominant modes of ocular shape variation and are ordered according to the amount of variance explained, quantified by the associated eigenvalues.

#### 5. Eye shape approximation.

Each eye can be approximated as the sum of the mean shape and a linear combination of *K Eigeneyes*:

(6) $$z_{n}\approx \bar{z}+\sum_{k=1}^{K}a_{k,n}e_{k},\;a_{k,n}=e_{k}^{⊤}\;\left(z_{n}-\bar{z}\right)$$

where $a_{k,n}$ represents the projection coefficient of eye *n* on the direction of the *k*-th *Eigeneye*, quantifying its deviation from the mean along that direction. Therefore, the 3-D geometry of each eye *n* is compactly represented by its vector of *Eigeneye* coefficients $a_{n}={\left[a_{1,n},\ldots ,a_{K,n}\right]}^{⊤}$, where *K* denotes the number of coefficients considered. In Section 3.1 we show that *K* = 6 provides a good trade-off between accuracy and compactness.

### 2.4 Synthetic data generation conditioned on age and axial length

The proposed framework enables modeling the *Eigeneye* coefficients and subsequently generating an arbitrary number of realistic synthetic eyes for a given *age* or/and *axial length* (AL), which are major sources of variability in eye geometry^3, 27^.

For a fixed value *x* of the conditioning variable *X* (i.e., age = *A* or AL = *L*), the *Eigeneye* coefficients ${\left\{a_{k}\right\}}_{k=1}^{K}$ were modeled as following a multivariate normal distribution:

(7) $$P\left(a_{1},\ldots ,a_{K}|X=x\right)∼𝒩\left(\mu_{x},\Sigma_{x}\right),$$

where *K* = 6 corresponds to the number of *Eigeneyes* considered.

**Figure 2.**
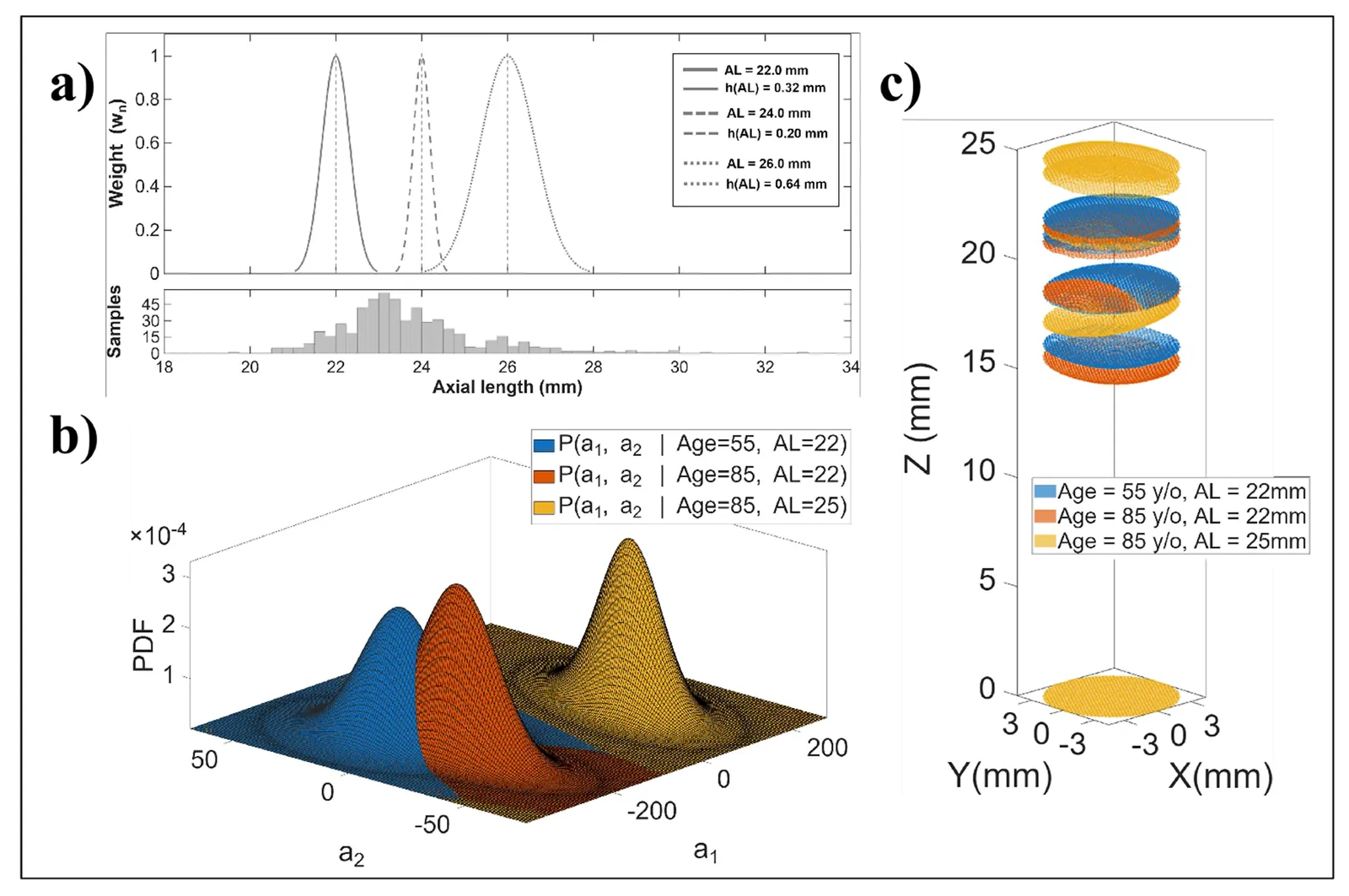
Illustration of the conditional *Eigeneyes* generation process. (a) Examples of adaptive Gaussian kernels for different target axial lengths, showing broader kernels in sparsely sampled regions and narrower kernels in densely sampled regions. Weights shown (*w_n_*) are not normalized for visualization purposes. (b) Estimated joint conditional probability density functions *P*(*a*_1_,*a*_2_ | Age, AL) for representative age and axial length combinations (c) Examples of synthetically generated ocular geometries obtained by sampling from the corresponding conditional distributions.

To estimate the conditional mean ***μ**_x_* and covariance Σ_*x*_, all samples were considered and weighted using a Gaussian kernel according to their proximity to the target value *x*^28^. Specifically, each sample *n* (i.e., *Eigeneye* coefficient vector $a_{n}={\left[a_{1,n},\ldots ,a_{K,n}\right]}^{⊤}$) was assigned a weight such that samples closer to *x* (i.e., **a**_*n*_ from eyes with age/AL closer to the target) had a greater influence, whereas more distant samples contributed less. However, since the distributions of age or AL were non-uniform in our dataset, some regions were densely sampled while others were sparsely represented. Using a fixed kernel bandwidth *h*_0_ would cause dense regions to dominate and sparse regions to be underrepresented, potentially biasing the estimates. To overcome this, an adaptive bandwidth for the Gaussian kernel *h* was used, resulting in narrower kernels in dense regions and broader kernels in sparse regions, producing stable and smooth estimates^29^. Figure 2a illustrates this idea by showing the Gaussian kernels used to assign relative weights to samples for different target axial lengths across the sample axial length distribution.

Formally, let *x_n_* denote the value of the conditioning variable for eye *n* (i.e., its age or AL). For a given target value *x*, each sample *n* was assigned a weight ${\tilde{w}}_{n}$ based on its proximity to *x* using a Gaussian kernel:

(8) $$w_{n}\left(x\right)=\exp\left[-\frac{{\left(x_{n}-x\right)}^{2}}{2h(x{)}^{2}}\right],\;{\tilde{w}}_{n}\left(x\right)=\frac{w_{n}\left(x\right)}{\sum_{j=1}^{N}w_{j}\left(x\right)},$$

with $\sum_{n=1}^{N}{\tilde{w}}_{n}(x)=1$. The bandwidth *h*(*x*) was target-dependent and inversely proportional to the estimated density of samples:

(9) $$h(x)=\frac{h_{0}}{f(x)}\cdot \frac{1}{\frac{1}{N}\sum_{i=1}^{N}\frac{1}{f\left(x_{i}\right)}},$$

where *f*(*x*) denotes the marginal probability density function of the conditioning variable evaluated at the target value, estimated using Gaussian kernel density estimation (KDE)^30^; *h*_0_ is a global bandwidth computed from Silverman’s rule^31^, scaling proportional to the data variability and decreasing with sample size $\left(h_{0}\propto \sigma_{X}N^{-1/5}\right)$; and the second term acts as a normalization factor.

The normalized weights ${\tilde{w}}_{n}(x)$ were then used to compute the conditional mean and covariance of the *Eigeneye* coefficients as

(10) $$\mu_{x}=\sum_{n=1}^{N}{\tilde{w}}_{n}a_{n},\;\Sigma_{x}=\sum_{n=1}^{N}{\tilde{w}}_{n}\left(a_{n}-\mu_{x}\right){\left(a_{n}-\mu_{x}\right)}^{⊤}.$$

Beyond conditioning on a single variable, the statistical eye model can also be jointly conditioned on both age and AL. For a target pair (*A*, *L*), the *Eigeneye* coefficients were assumed to follow a multivariate normal distribution conditioned on both variables,

(11) $$P\left(a_{1},\ldots ,a_{K}\;|\;\text{age}=A,\;AL=L\right)∼𝒩\left(\mu_{A,L},\Sigma_{A,L}\right).$$

The conditional mean ***μ***_*A*,*L*_ and covariance Σ_*A*,*L*_ were estimated from the complete dataset using an adaptive Gaussian kernel weighting scheme in the two-dimensional (age, AL) space. Under this approach, each sample received a weight of the form

$$w_{n}(A,L)=exp\left[-\left(\frac{{\left(A_{n}-A\right)}^{2}}{2h_{A}^{2}}\right)-\left(\frac{{\left(L_{n}-L\right)}^{2}}{2h_{L}^{2}}\right)\right],$$

where *h_A_* and *h_L_* denote the adaptive bandwidths for age and axial length, respectively. This weighting scheme jointly accounts for sample proximity and local data density. Beyond synthetic eye generation, this joint conditioning framework allows the analysis of age-related trends in geometrical and optical parameters for eyes with a given AL value (and vice versa), reducing confounding effects between global eye size and aging.

Once the conditional distribution of the coefficients had been estimated (conditioning on age = *A*, AL = *L*, or both), synthetic eye shapes were generated by sampling from these distributions to obtain an arbitrary number of *Eigeneye* coefficient vectors $\hat{a}$, which were then used to reconstruct the corresponding 3-D shapes using equation (6).

As an illustrative example of the process, Figure 2b shows the estimated joint conditional probability density functions of the coefficients *a*_1_ and *a*_2_ conditioned on age and axial length. Additionally, Figure 2c presents one example of a synthetically generated eye from each conditional distribution.

### 2.5 Data analysis

Quantitative morphological parameters were obtained from the eye models for subsequent analysis, specifically: curvature radii of the anterior and posterior surfaces of the cornea (RAC and RPC respectively) and the crystalline lens (RAL and RPL), defined as the best fitting sphere in a circular fitting area of 6 mm diameter; corneal thickness (CT); anterior chamber depth (ACD); vitreous chamber depth (VCD); lens thickness (LT); axial length (AL); and crystalline lens tilt magnitude (tilt_mag_) and direction (tilt_dir_), as defined in^22^. Furthermore, the optical powers of the cornea (CP), lens (LP), and total eye power (TP) were estimated using the paraxial thick lens formula^32^. The equivalent refractive index measured by Dubbelman^33^ was employed to model the lens refractive index.

To evaluate the *Eigeneyes* model representation accuracy and generalization performance (i.e., its ability to represent eyes not used in the model generation), a 10-fold cross-validation procedure was implemented. In each fold, the model was obtained using 90% of the dataset and evaluated on the remaining 10%, with the test subset rotated across folds. The entire 10-fold cross-validation procedure was repeated 100 times to reduce variability associated with random partitioning. Reconstruction accuracy was quantified using the root mean squared error (RMSE), computed as the average pointwise difference between each test eye and its reconstruction using *K Eigeneyes*. Reported RMSE values correspond to the mean error across all test samples and repetitions.

Normality of the data distributions was assessed using the Lilliefors test. When normality assumptions were not satisfied, non-parametric statistical analyses were used throughout the study.

Correlations between variables were assessed using Spearman’s rank correlation coefficient (***ρ***). Statistical significance was evaluated using a two-sided hypothesis test under the null hypothesis of zero correlation.

To determine whether differences in RMSE between the evaluated models were statistically significant, global differences were assessed using the Kruskal–Wallis test, followed by Bonferroni-corrected post-hoc pairwise comparisons.

Age-related and AL-related differences in biometric parameters were assessed using separate Kruskal–Wallis tests for each parameter, with Bonferroni correction applied to post-hoc pairwise comparisons.

Statistical significance was defined as *p* < 0.05 for all analyses. All statistical analyses were performed using MATLAB (MathWorks, Natick, MA, USA).

## 3 Results

### 3.1 Selection of the number of *Eigeneyes*

The selection of the optimal number of *Eigeneyes*, *K*, is important to achieve a balance between model compactness and an accurate representation of the eye geometry.

To estimate the optimal value of *K*, two complementary metrics were considered. The first was the percentage of explained variance (PVar), which quantifies the proportion of geometrical variability captured by the first *K Eigeneyes*. The second was the RMSE, which measures the average reconstruction error between the original shapes and those reconstructed using the first *K Eigeneyes*. Both metrics were evaluated using 10-fold cross validation procedure repeated 100 times. The mean and standard deviation of PVar and RMSE were calculated in the test sets across the 100 repetitions.

**Figure 3.**
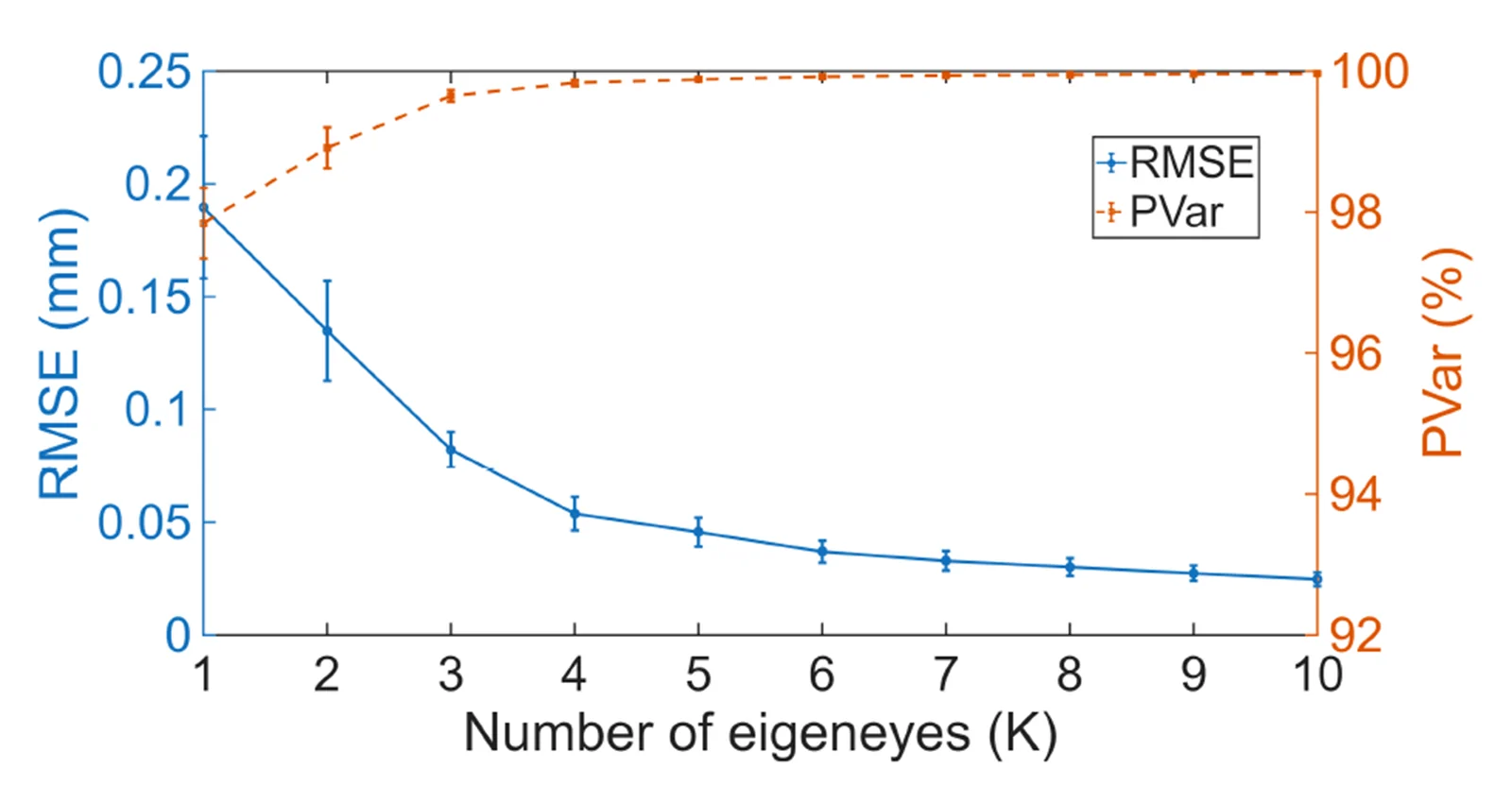
Reconstruction performance. RMSE and percentage of variance explained (PVar) as a function of the number of *Eigeneyes* used in the representation.

The results in Figure 3 show that, as expected, the RMSE decreases when increasing the number of *Eigeneyes* while PVar increases up to *K* = 10, after which it stabilizes. *K* = 6 provides a good trade-off between accuracy and model complexity, yielding an average reconstruction error below 0.035 mm and an explained variance above 99%. Consequently, *K* = 6 was selected as the optimal value and is used in all subsequent analyses.

### 3.2 *Eigeneyes* description

Each *Eigeneye* **e**_*k*_ was visually and quantitatively analyzed to characterize the morphological variations it represents. Controlled deformations were applied to the mean eye shape $\bar{z}$ along each *Eigeneye* direction using coefficients of ±3***σ***_*k*_, where ***σ***_*k*_ denotes the standard deviation of the projection coefficients *a*_*k*,*n*_:

(12) $$z_{\left(\pm 3\sigma_{k}\right)}=\bar{z}\pm 3\sigma_{k}e_{k}.$$

**Figure 4.**
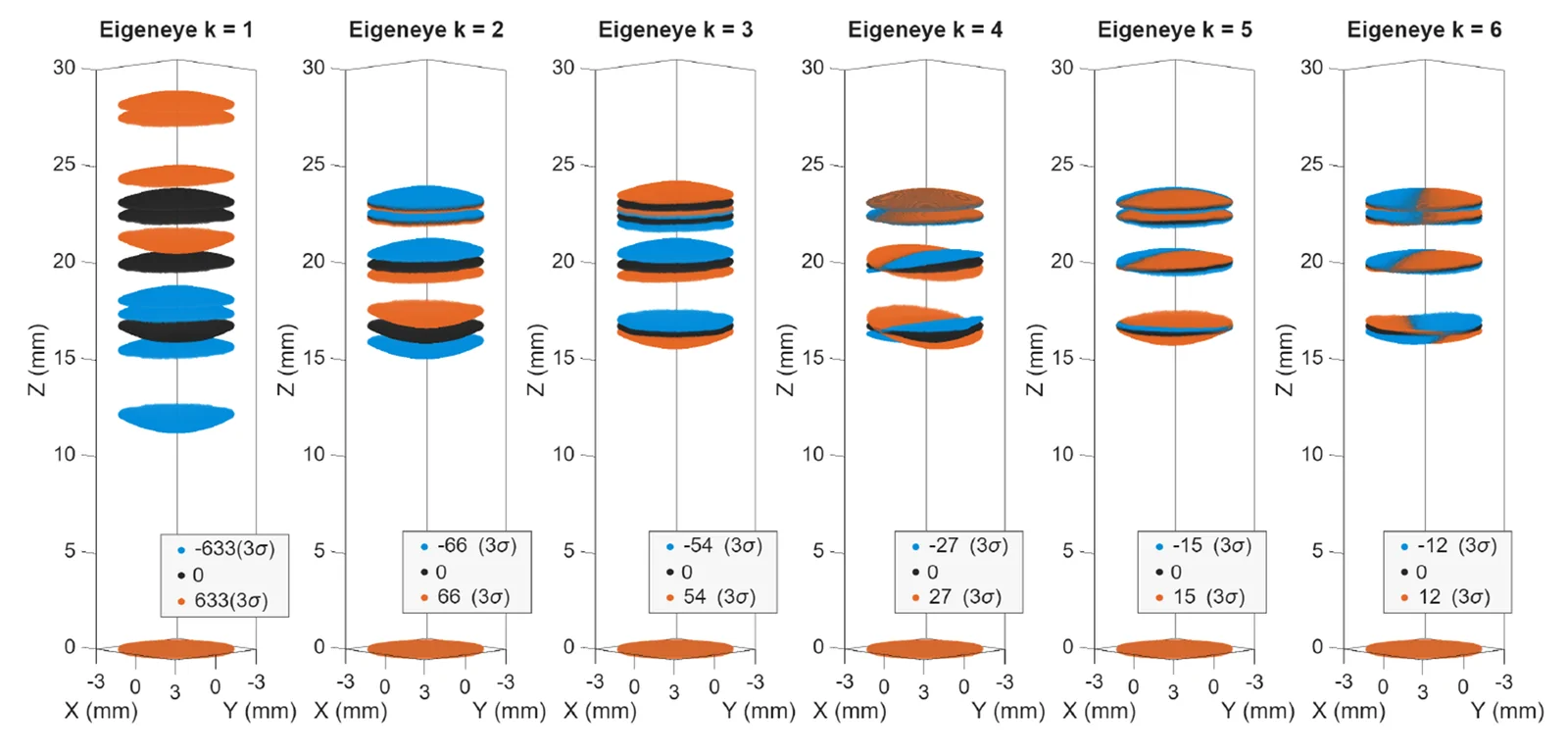
Representation of the first six *Eigeneyes*. For each coefficient *a_k_*, three values were evaluated: −3***σ***_*k*_ (blue), 0 (black), and +3***σ***_*k*_ (orange), where ***σ***_*k*_ denotes the standard deviation of the coefficients associated with *Eigeneye k* across the full dataset. The mean eye shape (*a_k_* = 0) is shown in black in all subfigures. For each color-coded eye, the different ocular surfaces are displayed (i.e., from top to bottom, anterior corneal surface, posterior corneal surface, anterior crystalline lens surface, posterior crystalline lens surface, and retina).

Following this procedure, synthetic eyes that represent realistic extremes of shape variations for each *Eigeneye* were generated. Several biometric parameters were quantified from these shapes, including RAC, RPC, RAL, RPL, CT, LT, ACD, VCD and AL. For each parameter *P*, the influence of *Eigeneye k* was quantified by computing the peak-to-peak variation across the deformation range, $\Delta P=P_{max}-P_{min}$. Figure 4. shows the geometrical changes in the generated eyes from the first 6 *Eigeneyes*. A quantitative summary of the main changes induced by each *Eigeneye* is provided in Table 1.

Each *Eigeneye* represents a global deformation of the average ocular geometry, with lower-order modes primarily governing large-scale axial variations and higher-order modes encoding more localized geometric refinements.

The first *Eigeneye* (**e**_1_) captures global axial scaling, producing large variations in AL mainly driven by changes in VCD and ACD, together with coordinated curvature adjustments of both the cornea and crystalline lens. The second and third *Eigeneyes* (**e**_2_ and **e**_3_) are primarily associated with lens geometry, with **e**_2_ affecting lens thickness and shape, and **e**_3_ encoding changes in lens curvature and position, particularly reflected in ACD.

Higher-order components (**e**_4_–**e**_6_) account for smaller-scale deformations, including lens tilt and localized curvature asymmetries, while having negligible impact on global axial dimensions. Although their contribution to overall shape variation is smaller, these modes play an important role in refining anatomical detail and capturing inter-individual variability.

An interactive application was developed to facilitate the interpretation of the *Eigeneyes*, allowing visualization of the geometric changes associated with each coefficient and the resulting full-eye shape generated from their combination. The application is available upon request, and a demonstration video is included in the Supplementary Video S1.

### 3.3 Correlation of *Eigeneye* coefficients with age and AL

The second *Eigeneye* coefficient, **e**_2_, showed a moderate negative correlation with age (***ρ*** = −0.50, *p*-value < 0.05, slope = −0.79 years^−1^), suggesting that **e**_2_ is sensitive to structural changes associated with aging, likely reflecting variations in crystalline lens geometry. Weaker but still statistically significant correlations with age were also found for the first *Eigeneye* coefficient, **e**_1_, (***ρ*** = −0.18, *p* < 0.05), for the third *Eigeneye* coefficient, **e**_3_, (***ρ*** = −0.10, *p* < 0.05) and for the sixth *Eigeneye* coefficient **e**_6_, (***ρ*** = −0.10, *p* < 0.05).

In contrast, AL showed a strong and statistically significant association with the first *Eigeneye* coefficient, **e**_1_, (***ρ*** = 0.93, *p*-value < 0.05, slope = 119.68 mm^−1^). This strong linear relationship supports the interpretation of **e**_1_ as primarily encoding AL.

**Figure 5.**
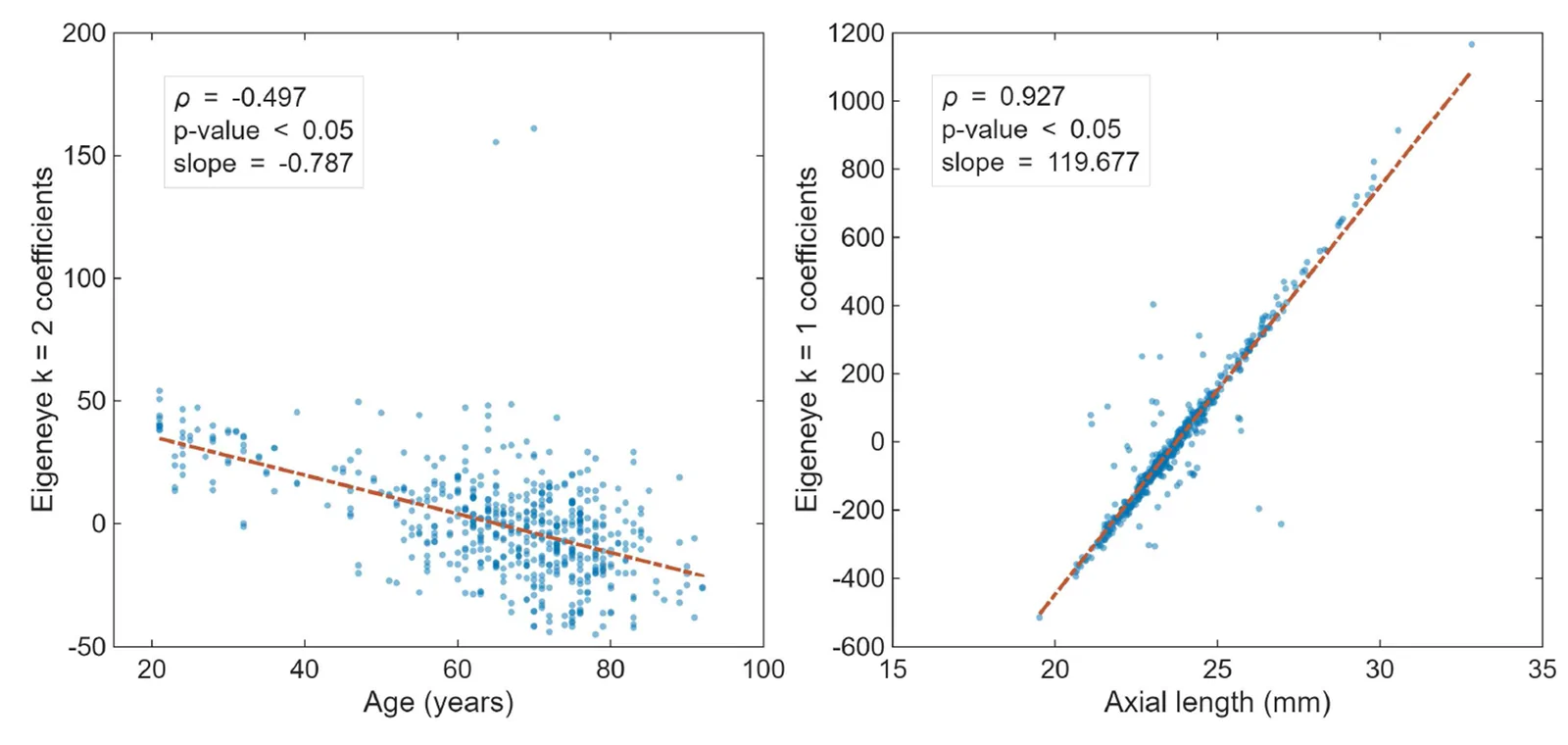
Most relevant statistically significant correlations (*p* < 0.05) between *Eigeneyes* coefficients and age or AL. Each subplot displays the linear regression fit together with the Spearman correlation coefficient (***ρ***), *p*-value, and regression slope.

Figure 5 summarizes the most relevant statistically significant relationships between the first six *Eigeneyes* coefficients and both age and AL, including the corresponding linear regression slopes, correlation coefficients, and *p*-values.

### 3.4 Comparison with other mathematical models

The representation ability of the *Eigeneyes* was compared with that of two conventional geometric models that rely on a reduced set of biometric parameters commonly used to describe ocular geometry^34^.

The first description, referred to as the 8-parameter method (8-PAR), consists of eight geometric parameters: RAC, RPC, RAL, RPL, CT, LT, ACD and VCD. The second, referred to as the 16-parameter method (16-PAR), extends the 8-PAR formulation by additionally including the *x* and *y* coordinates of the best fitting sphere center of each ocular surface, resulting in a total of 16 geometric parameters. This extension allows surface decentrations and tilts to be explicitly represented within the model. Also, the RMSE was obtained considering a different number of *Eigeneyes* (EE) coefficients (four, six and twelve coefficients).

**Figure 6.**
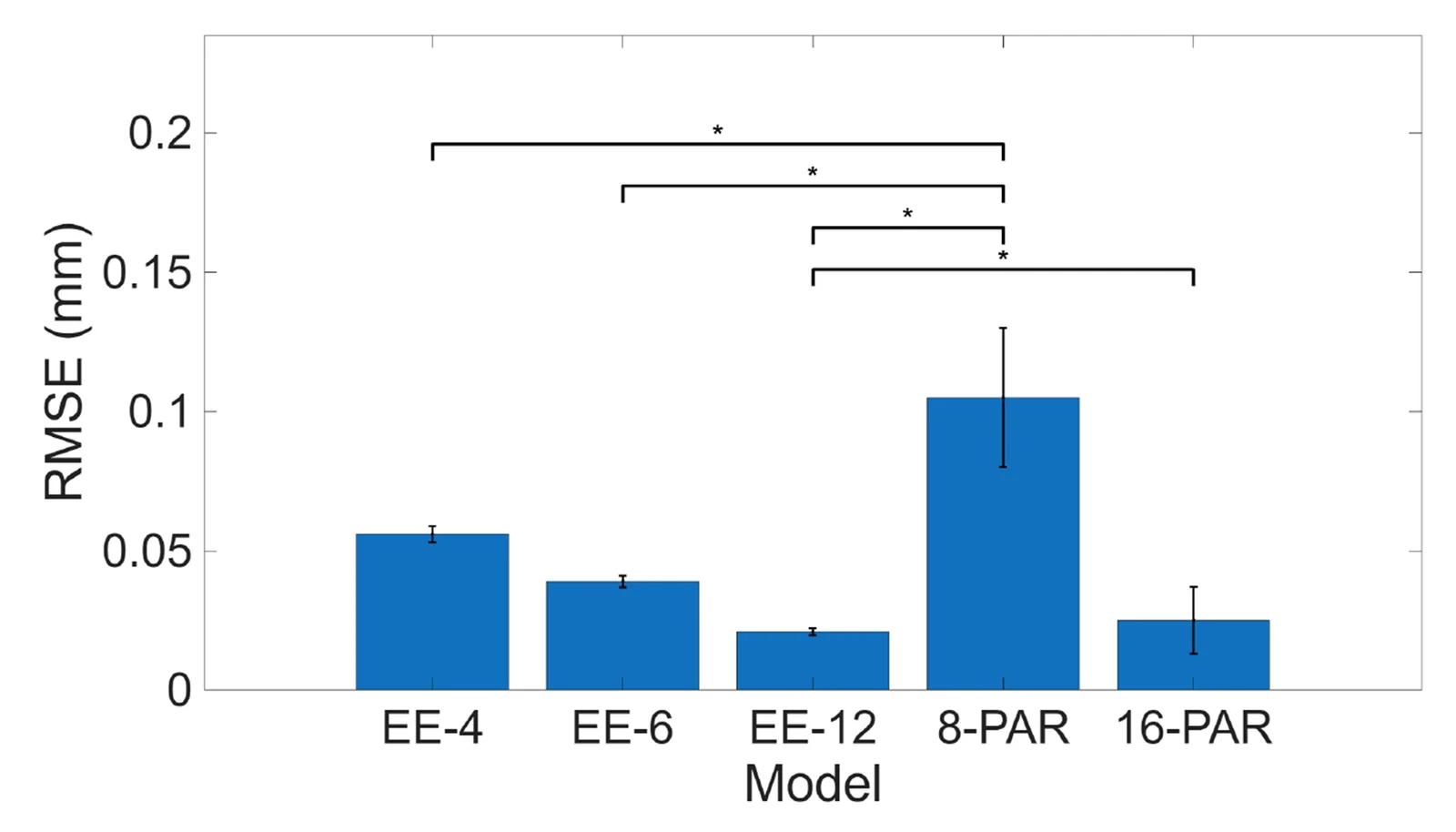
RMSE comparison for different ocular geometry representation methods: *Eigeneyes* (EE) using 4, 6 and 12 principal components, and two classical geometric descriptions of the anterior segment, namely the 8-parameter (8-PAR) and 16-parameter (16-PAR) models. Error bars represent the standard deviation across test eyes. Asterisks denote statistically significant pairwise differences obtained from Kruskal–Wallis test with Bonferroni-corrected post-hoc multiple comparisons (*p* < 0.05).

Figure 6 illustrates the comparative performance of the different representations in the test set of data (cross validation). The mean RMSE were 0.056 ± 0.003 mm, 0.039 ± 0.002 mm and 0.021 ± 0.001 mm using four, six and twelve *Eigeneyes* respectively.

The 8-PAR model produced a significantly higher mean RMSE (0.105 ± 0.026 mm) than all *Eigeneyes* representations ((Kruskal–Wallis test with Bonferroni-corrected post-hoc multiple comparisons, *p* < 0.05). Notably, the model using twelve *Eigeneye* coefficients significantly outperformed the classical 16-PAR model (0.025 ± 0.016 mm; Bonferroni-corrected Wilcoxon signed-rank post-hoc comparison, *p* < 0.05).

These results demonstrate that the *Eigeneyes* provides a more compact and accurate representation of the eye geometry than traditional parametric models, achieving superior reconstruction accuracy with fewer parameters.

### 3.5 Analysis of synthetic eye data conditioned on age and axial length

1000 synthetic eyes were generated using the proposed statistical framework, jointly conditioned on age (30, 55, and 80 years) and AL (22, 24, and 26 mm). To quantify variations with age and AL, a set of geometrical and optical variables was calculated from each synthetic eye, namely: RAC, RPC, RAL, RPL, ACD, VCD, LT, CT, crystalline lens tilt magnitude (tilt_mag_) and direction (tilt_dir_), as well as corneal, lenticular, and total ocular power (CP, LP and TP respectively).

**Figure 7.**
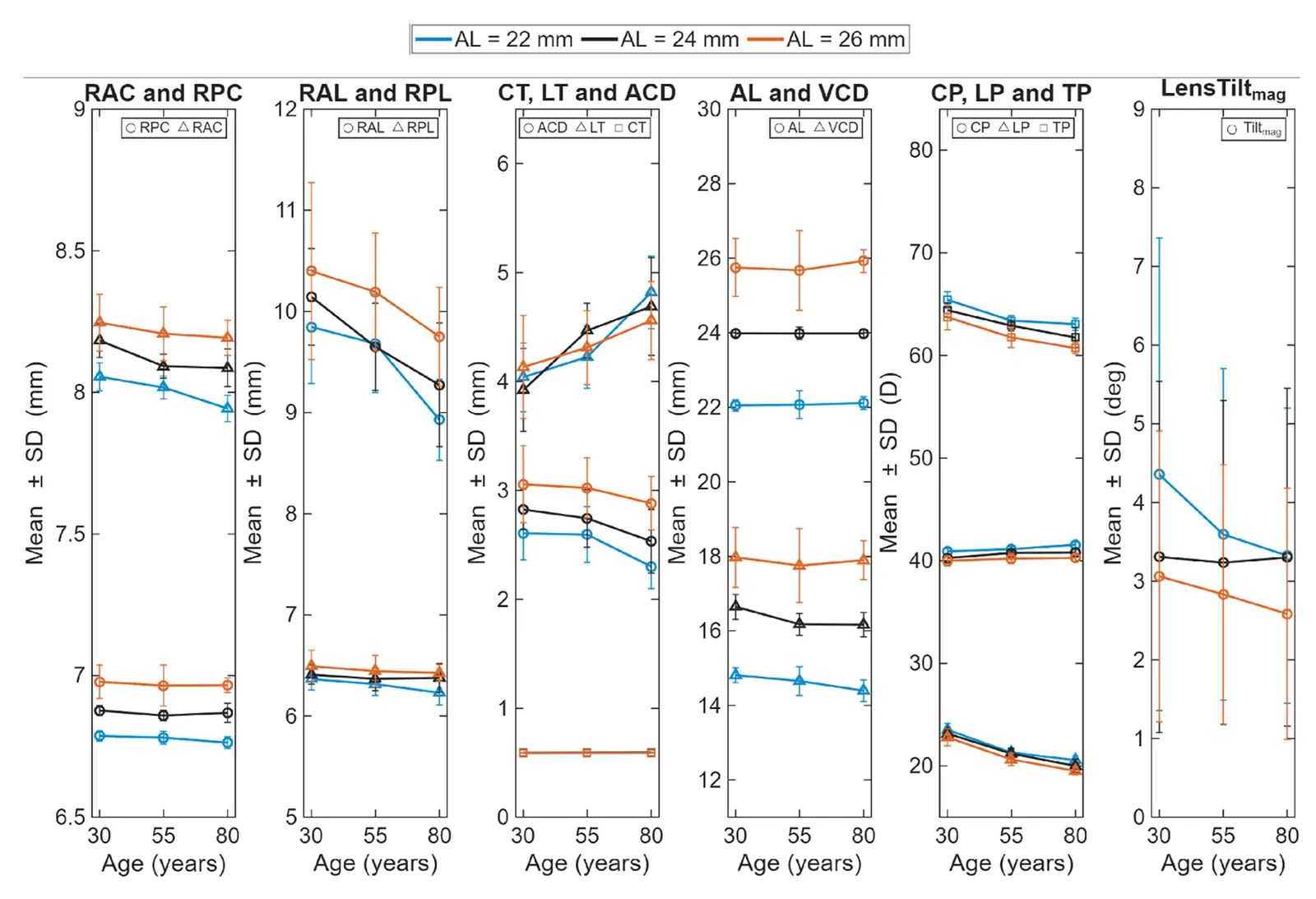
Variations of biometric and optical parameters for 1000 synthetic eyes generated under fixed axial length conditions (22, 24 and 26 mm) and age (30, 55 and 80 years). Mean values are shown for corneal radii (RPC, RAC), lens radii (RAL, RPL), corneal thickness (CT), anterior chamber depth (ACD), axial length (AL), vitreous chamber depth (VCD), lens power (LP), total power (TP) and lens tilt magnitude. Changes in AL are represented by differences between curves of different colors, while changes with age are reflected by the slope of each individual curve.

Figure 7 summarizes biometric and optical variations at fixed AL (22, 24, and 26 mm; blue, black, and red curves, respectively) across age (x-axis of each panel). Thus, differences between curves quantify the effect of AL, whereas the slope within each curve reflects aging effects. Unless otherwise stated, the trends described below correspond to statistically significant differences (Kruskal-Wallis test with Bonferroni-corrected post hoc pairwise comparisons, *p* < 0.05).

When age was fixed (30, 55, or 80 years), AL effects were strong and monotonic for several parameters. VCD was predominantly driven by AL, showing a clear separation between groups at all ages (e.g., at 30 years: 14.81 ± 0.20 mm for *AL* = 22 mm vs 17.97 ± 0.80 mm for *AL* = 26 mm). ACD also increased with AL (e.g., at 30 years: 2.60 ± 0.24 mm at*AL* = 22 mm to 3.05 ± 0.35 mm at *AL* = 26 mm). Corneal geometry followed the same trend, with RAC and RPC increasing with AL (flatter corneas in longer eyes), leading to a decrease in corneal power (CP) (e.g., at 30 years: 41.90 ± 0.25 D at *AL* = 22 mm to 40.93 ± 0.49 D at*AL* = 26 mm). Finally, lens RAL also increased with AL for a given age (e.g., at 30 years: 9.84 ± 0.55 mm at *AL* = 22 mm vs 10.39 ± 0.87 mm at *AL* = 26 mm).

When AL was fixed, aging effects became more evident and were largely consistent across AL groups. Lens geometry showed a clear age dependence, with RAL decreasing from 30 to 80 years (e.g., at*AL* = 22 mm: 9.84 ± 0.55 mm to 8.92 ± 0.40 mm). This was accompanied by a strong decrease in lens power (LP) (e.g., at *AL* = 22 mm: 23.52 ± 0.61 D at 30 years, 21.28 ± 0.44 D at 55 years, and 20.56 ± 0.42 D at 80 years), resulting in a parallel reduction in total ocular power (TP). Geometrically, aging was characterized by a marked decrease in ACD and an increase in LT across all AL groups (e.g., at *AL* = 24 mm: ACD from 2.82 ± 0.18 mm at 30 years to 2.53 ± 0.29 mm at 80 years; LT from 3.92 ± 0.38 mm to 4.68 ± 0.44 mm). In contrast, VCD exhibited only modest age-related changes once AL was controlled (e.g., at AL = 22 mm: 14.81 ± 0.20 mm at 30 years to 14.39 ± 0.29 mm at 80 years), confirming that posterior elongation is primarily driven by AL rather than aging.

The lens tilt magnitude showed a combined dependence on both age and AL, decreasing systematically with increasing AL within each age group (e.g., at 30 years: 4.36 ± 3.01° at AL = 22 mm to 3.06 ± 1.85° at AL = 26 mm), while also exhibiting a mild age-related reduction at fixed AL (e.g., at AL = 22 mm: 4.36 ± 3.01° at 30 years to 3.32 ± 1.88° at 80 years).

## 4 Discussion

The *Eigeneyes* framework could be understood as a natural extension of statistical shape modeling approaches previously applied to individual surfaces of the eye^17, 20^, generalizing this concept to the complete three-dimensional geometry of the eye. Modeling the whole eye provides important advantages. In particular, *Eigeneyes* captures coupled geometric variations across multiple structures, reflecting the intrinsic interdependence between ocular components that arises from coordinated growth and biomechanical constraints during ocular development^35^. This unified representation enables the analysis of coordinated shape changes that cannot be observed when structures are treated independently. Furthermore, the statistical treatment of the model coefficients allows the generation of anatomically plausible synthetic eyes.

A total of 1000 synthetic eyes were generated using *Eigeneyes* and subsequently characterized in terms of their biometric and optical properties. Conditioning jointly on age and AL allowed us to study the independent effects of both variables. The age-conditioned analysis reproduced the main trends reported in the literature. In particular, in the range between 30 and 80 years, aging was associated with a gradual shallowing of the anterior chamber, an increase in lens thickness, a steepening of the anterior lens surface, and a reduction in lens power. Quantitatively, ACD decreased by approximately 0.49 mm between 30 and 80 years, while LT increased by about 1.01 mm over the same range. These values were in good agreement with in vivo studies reporting age-related decreases in ACD and increases in LT^33, 36^, with typical rates of change of around −0.01 to −0.02 mm/year for ACD and +0.02 to +0.03 mm/year for LT^33, 37^. Similarly, the pronounced decrease in RAL observed in our synthetic eyes, together with the smaller change in RPL, is consistent with previous findings showing that the anterior lens surface undergoes stronger age-related changes than the posterior surface^33, 36^. The reduction in LP is also in line with studies in adult populations, where lens power decreases with age and tends to level off in older subjects^38^.

In addition to these geometric and optical changes, our model revealed a mild reduction in crystalline lens tilt magnitude with age for fixed axial length. By contrast, corneal changes in our model were comparatively small: CP increased only slightly with age, which is compatible with the general view that corneal geometry is relatively stable across adulthood^33, 36^, although mild age-related steepening has been reported in some populations^39^. Overall, these comparisons support that the dominant aging signature captured by the model is lens-driven rather than cornea-driven, with additional secondary effects on lens alignment.

The AL-conditioned analysis also agrees with the state of the art, showing that longer eyes are primarily characterized by posterior segment elongation, deeper anterior chambers, flatter corneas and lenses, and lower corneal and total power. In our synthetic eyes, VCD and ACD increased monotonically with AL, while RAC, RPC and RAL increased and CP decreased, indicating progressive corneal and lens flattening in longer eyes. This pattern matches previous biometric studies showing that AL is positively associated with ACD and negatively associated with corneal curvature^40, 41^. The reduction in LP with increasing AL is also consistent with reports in both children and adults indicating lower lens power in more myopic or longer eyes^38, 42^. Also, lens tilt showed a clear and systematic decrease with increasing AL, which is in agreement with recent swept-source OCT studies reporting reduced tilt magnitude in longer eyes^22, 43^.

Another advantage of the proposed method is that it allows sampling at any age or AL, facilitating fair comparisons between studies. Differences in sampling or in the ranges considered can lead to variations in correlation values or slopes, making direct comparisons difficult.

Gaussianity was assumed for the conditional probability density functions of the *Eigeneyes* coefficients. To assess this assumption, multivariate normality was evaluated using Mardia’s skewness and kurtosis tests^44^. The results indicated no significant deviations from multivariate normality, supporting the use of a Gaussian model.

The distributions of the coefficients conditioned on age and AL were analyzed in the paper, as these represent the two major sources of variability in eye geometry. Nevertheless, other types of conditioning could be explored. For example, we generated 2000 eyes conditioned on laterality (i.e., 1000 left and 1000 right eyes). The tilt direction for left eyes was 160.77° ± 28.87°, whereas for right eyes it was 17.47° ± 32.97°, which means nasal tilt for both eyes (see Supplementary Figure S1). These results are consistent with tilt directions reported in previous studies^22, 45, 46^. When analyzed the coefficient statistics, we found that the coefficient expressing the lens tilt direction (*a*_4_) changes its sign (from values of −7.3 ± 4.9 for right eyes to around 8.4 ±4.9 for left eyes), reflecting the mirror-symmetric tilt direction between left and right eyes. We also examined conditioning based on sex, comparing male and female eyes, and found that the model captures expected biometric differences^47^, with males presenting longer axial lengths (23.89 ± 1.38 mm vs 23.56 ± 2.04 mm) and females showing higher corneal power (40.86 ± 0.76 D vs 40.72 ± 0.60 D). In addition, males exhibited slightly larger anterior lens radii (9.77 ± 0.85 mm vs 9.40 ± 0.80 mm), indicating a flatter anterior lens surface, consistent with their overall longer eye geometry. All these options can be explored in the online app (see Supplementary Video S2), which allows users to generate eye models for a given age, AL, sex, or eye laterality.

This work focuses on obtaining a geometrical representation of the entire eye over a wide area (in this case, 6 mm, which corresponds to the maximum diameter measurable with the IOLMaster 700). Zernike smoothing with the first six coefficients was applied in the crystalline lens, allowing robust extrapolation into the crystalline lens surfaces, particularly for eyes with small pupils. Nevertheless, this approach precludes the incorporation of lens asphericity. We also derived the *Eigeneyes* restricting the analysis to 3 mm pupils (i.e., without extrapolation of the lens surfaces) using the spherical aberration Zernike term in addition to the 6 lower order coefficients. The *Eigeneyes* representation remained practically unchanged. These results are presented in the supplementary material (see Supplementary Figure S2). While a 3 mm pupil allows a more accurate description of the lens surfaces in the paraxial area, which is advantageous for applications such as power calculation, a 6 mm diameter is more convenient for other purposes, such as intraocular lens (IOL) position estimation in cataract surgery or the study of lens remodeling in myopia. The extrapolation area and number of Zernike coefficients used in the extrapolation can be selected in the online app (see Supplementary Video S2).

In this work, we selected six *Eigeneyes* as the optimal representation. Nevertheless, if a particular application requires a more accurate description of the ocular surfaces (e.g., to capture higher-order corneal surface deformations), this can be easily achieved by increasing the number of *Eigeneyes* included in the model. As a preliminary analysis, we evaluated the correlations between astigmatism ($Z_{2}^{-2}$ and $Z_{2}^{2}$) and spherical aberration ($Z_{4}^{0}$) Zernike coefficients of the different ocular surfaces and the *Eigeneye* coefficients. Several statistically significant and relatively strong correlations were observed, of which only a few representative examples are reported here (where *eign* denotes the coefficient associated with the *n*-th *Eigeneye* mode, ordered according to the variance explained): $\rho_{Z_{2}^{-2}-eig31}=0.69$ and $\rho_{Z_{4}^{0}-eig35}=0.65$ for the anterior corneal surface, $\rho_{Z_{2}^{-2}-eig27}=0.62$ for the posterior corneal surface, $\rho_{Z_{2}^{-2}-eig17}=-0.69$ for the anterior lens surface, and $\rho_{Z_{2}^{2}-eig9}=0.89$ for the posterior lens surface. A detailed analysis of the relationship between higher-order ocular aberrations and the *Eigeneye* coefficients, as well as the selection of the optimal number of *Eigeneyes* for specific applications, is left for future work.

A potential limitation of this study is the inclusion of both eyes from the same participant, which could potentially introduce inter-eye correlations and bias the model. To assess the impact of this effect, we constructed the *Eigeneye* models using a subset of the data that included only one eye per participant. The resulting *Eigeneyes* showed only minor differences compared with those derived from the full dataset including both eyes.

Beyond methodological considerations, the main strength of the*Eigeneyes* framework lies in its global representation of ocular geometry. Unlike traditional schematic or parametric eye models that define each ocular surface independently through predefined geometric parameters^32^, the proposed model captures the complete three-dimensional geometry of the eye as a single, coupled system. This formulation naturally preserves the geometric relations between ocular components such as the cornea, crystalline lens, and AL, leading to anatomically consistent eye shapes avoiding unrealistic parameter combination that may arise when separate models derived from different datasets are combined.

This complete and compact geometric description is particularly relevant for applications such as cataract surgery planning. Accurate estimation of the lens position after crystalline lens removal is essential for precise intraocular lens (IOL) power calculation. Over the years, IOL power calculation formulas have increasingly recognized the importance of anterior segment geometry and its interaction with axial length in predicting postoperative outcomes^48^. The compactness of the *Eigeneyes* provides an advantage for estimation problems, for example when using machine learning approaches^49^.

Beyond surgical planning, the ability to model coordinated whole-eye variations also supports the study of refractive development and aging. Both ocular growth and aging processes involve distributed structural changes affecting multiple components simultaneously, including axial elongation, anterior chamber depth variation, and lens remodeling^1, 3, 50^.

Overall, these results suggest that *Eigeneye* representations enables new possibilities for anatomical analysis, optical modeling, and personalized eye representation.

## 5 Conclusion

The *Eigeneye* concept has been introduced in this work, which is a statistical model that provides a compact and accurate representation of the complete three dimensional geometry of the human eye.

This work demonstrates that *Eigeneyes* achieve significantly higher reconstruction accuracy with lower parameters than other parametric methods while maintaining interpretability. Realistic synthetic eyes are generated thanks to the probabilistic formulation of the model, which also enables conditioning of ocular geometry on biometric variables such as age and axial length. This capability allows aging effects to be disentangled from axial phenotype in a physiologically meaningful manner.

These abilities make the *Eigeneyes* framework a powerful tool for personalized eye modeling, with potential applications in optical simulation, surgical planning, and the study of refractive conditions and age related ocular changes.

## Supplementary Material

This is a list of supplementary files associated with this preprint. Click to download.

Videoapp1.mov

Videoapp1.mov

Videoapp1.mov

VideoAPP2.mov

VideoAPP2.mov

VideoAPP2.mov

SUPPLEMENTARYEIGENEYES.pdf

SUPPLEMENTARYEIGENEYES.pdf

SUPPLEMENTARYEIGENEYES.pdf

LaTeXsupplementaryfile.bbl

LaTeXsupplementaryfile.bbl

LaTeXsupplementaryfile.bbl

**Supplementary information** accompanies this paper at.

## Figures and Tables

**Table 1.** Main geometric variations induced by each *Eigeneye* when varying its coefficient within ±3***σ***_*k*_. Peak-to-peak variations (Δ*P*, in mm or degrees) are reported.

*Eigeneye*	Main effect	Key variations (Δ*P*)
**e**_1_	Global axial scaling	AL: 10.07 mm VCD: 9.24 mm ACD: 1.33 mm RAC: 0.67 mm RPC: 0.65 mm RAL: 2.08 mm
**e**_2_	Lens thickness and shape	LT: 2.85 mm RAL: 3.35 mm ACD: 1.01 mm RAC: 0.49 mm RPL: 0.50 mm
**e**_3_	Lens curvature and position	RAL: 2.98 mm ACD: 1.95 mm
**e**_4_	Lens tilt direction	tilt_dir_: 171.3°
**e**_5_	Lens tilt magnitude	tilt_mag_: 1.69°
**e**_6_	Cornea and lens curvature	RAC: 0.18 mm RPC: 0.04 mm RAL: 0.34 mm tilt_mag_: 1.67°

## Data Availability

The datasets analysed during the current study are available in a GitHub repository: https://github.com/EstelaVadillo17/Eigeneyes-A-statistical-model-of-whole-eye-geometry-and-its-applications.git
